# Long-term mortality and outcome in hospital survivors of septic shock, sepsis, and severe infections: The importance of aftercare

**DOI:** 10.1371/journal.pone.0228952

**Published:** 2020-02-12

**Authors:** Tim Rahmel, Stefanie Schmitz, Hartmuth Nowak, Kaspar Schepanek, Lars Bergmann, Peter Halberstadt, Stefan Hörter, Jürgen Peters, Michael Adamzik

**Affiliations:** 1 Klinik für Anästhesiologie, Intensivmedizin und Schmerztherapie, Universitätsklinikum Knappschaftskrankenhaus Bochum, Bochum, Germany; 2 Institut für Versorgungsforschung der Knappschaft, Knappschaft, Bochum, Germany; 3 Klinik für Anästhesiologie und Intensivmedizin, Universität Duisburg-Essen & Universitätsklinikum Essen, Essen, Germany; Azienda Ospedaliero Universitaria Careggi, ITALY

## Abstract

Patients with severe infections and especially sepsis have a high in-hospital mortality, but even hospital survivors face long-term sequelae, decreased health-related quality of life, and high risk of death, suggesting a great need for specialized aftercare. However, data regarding a potential benefit of post-discharge rehabilitation in these patients are scarce. In this retrospective matched cohort study the claim data of a large German statutory health care insurer was analyzed. 83,974 hospital survivors having suffered from septic shock, sepsis, and severe infections within the years 2009–2016 were identified using an ICD abstraction strategy closely matched to the current Sepsis-3 definition. Cases were analyzed and compared with their matched pairs to determine their 5-year mortality and the impact of post-discharge rehabilitation. Five years after hospital discharge, mortality of initial hospital survivors were still increased after septic shock (HR_adj_ 2.03, 95%-CI 1.87 to 2.19; *P*<0.001), sepsis (HR_adj_ 1.73, 95%-CI 1.71 to 1.76; *P*<0.001), and also in survivors of severe infections without organ dysfunction (HR_adj_ 1.70, 95%-CI 1.65 to 1.74; *P*<0.001) compared to matched controls without infectious diseases. Strikingly, patients treated in rehabilitation facilities showed a significantly improved 5-year survival after suffering from sepsis or septic shock (HR_adj_ 0.81, 95%-CI 0.77 to 0.85; *P*<0.001) as well as severe infections without organ dysfunction (HR_adj_ 0.81, 95%-CI 0.73 to 0.90; *P*<0.001) compared to matched patients discharged to home or self-care. Long-term mortality and morbidity of hospital survivors are markedly increased after septic shock, sepsis and severe infections without organ dysfunction, but best 5-year survival was recorded in patients discharged to a rehabilitation facility in all three groups. Thus, our data suggest that specialized aftercare programs may help to improve long-term outcome in these patients and warrants more vigilance in future investigations.

## Introduction

Severe infections are a global health care challenge with an increasing incidence and a tremendous impact on health-care costs and mortality [1–3]. Recent estimates of the incidence of severe infections in the intensive care unit (ICU) consider that more than 50% of the ICU patients are infected and more than 70% receive antibiotics [4, 5].

Since the latest update of the sepsis-definition by consensus, severe infections are stratified as infections without organ dysfunction, sepsis, and septic shock [6], and this is believed to allow a reliable risk stratification of these patients regarding their in-hospital mortality [7]. However, sequelae and long-term outcome may not only relate to acute organ dysfunction, but rather to a “dysregulated”, long lasting host immune response that may explain the high mortality with severe infections [4, 8]. Furthermore, due to the progress in intensive care medicine the absolute number of survivors to hospital discharge increases and should be expected to increase further [1, 2]. Accordingly, it appears prudent to assess beyond hospital discharge their long-term prognosis and medical burden [9–11].

In the context of sepsis, prior studies demonstrated a persistently impaired quality of life and increased long-term mortality [9, 12]. Additionally, initial survivors demonstrated a markedly increased healthcare usage and an increased mortality when compared with a matched cohort of other hospitalized patients [13]. Furthermore, a worldwide survey of sepsis survivors found that respondents suffered from many physiologic, physical, and psychological impairments covering virtually every organ system and ability [14]. This suggests that there is an enormous need for specific aftercare management programs, post-discharge treatment strategies, and rehabilitation concepts so as to deal with the long-term consequences. However, to our knowledge, large studies exploring a potential impact of post-discharge rehabilitation on long-term outcome of severe infections and especially sepsis are widely lacking [11, 15].

Consequently, we assessed 3-month to 5-year mortality, most common medical sequelae, and the impact of post-discharge rehabilitation after hospital discharge in survivors of former severe infections without organ dysfunction, sepsis and septic shock and classified these patients using an ICD abstraction strategy closely matched to the Sepsis-3 definition.

## Methods and methods

Using a retrospective cohort design, we estimated the 5-year post-discharge mortality, new health related diagnoses after hospital discharge, discharge destination from the index hospital admission episode, and degree of care dependency one year after index hospital admission compared with the degree within one year prior to the index admission. This study was reviewed and approved by the Ethics Committee of the Medical Faculty of the University of Bochum (no. 18–6470). Because of the deidentified nature of the analyzed data the requirement for informed consent was waived.

### Patients and sample selection

**Fig 1:**
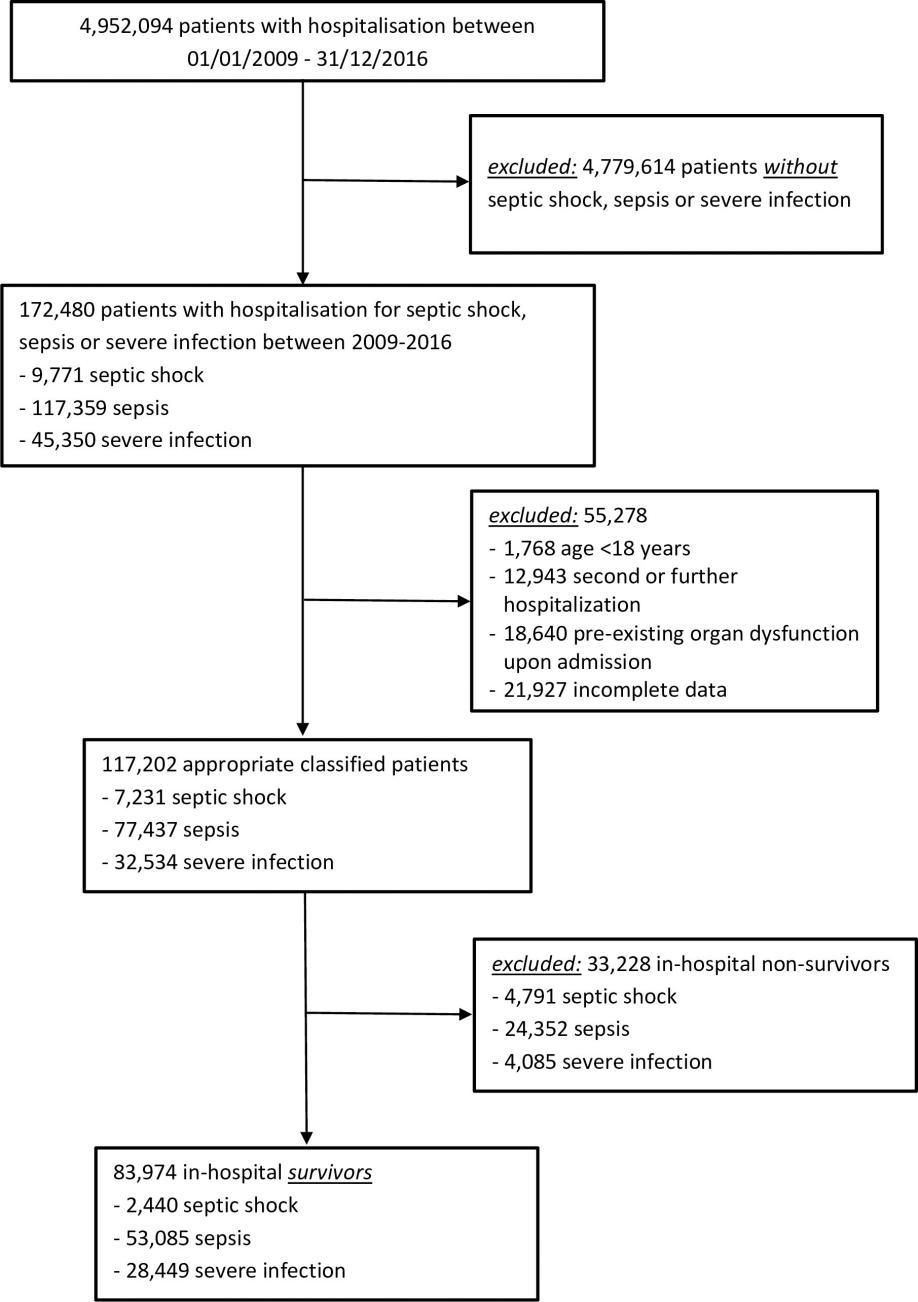
Flow chart showing selection of severe infections, sepsis, and septic shock patients stratified for in-hospital survivors and non-survivors.

Patients’ data were obtained from a large German health care insurance claims database spanning the time period from January 1, 2009, through June 30, 2018. The study sample and the matched control group consisted of patients that had been admitted to an intensive care unit and the inpatient claim had indicated the presence of an infection up to December 31, 2016 (Fig 1) or no infection, respectively.

Eligible cases were identified by using the International Classification of Diseases, 10th revision, German modification (ICD-10-GM) codes, according to infection codes and explicit sepsis codes (see S1 Table), as described previously [16–18]. If an individual patient had more than one hospital admission between January 1, 2009, and December 31, 2016, only the first index admission was considered eligible. Patients with pre-existing end-stage renal disease, home respiratory care, severe dementia, or a delirium 12 month before the index were identified using related ICD or OPS encodings. These patients were excluded from analysis to minimize a false positive patient selection due to confounding pre-existing organ dysfunctions in appreciation of the current sepsis-3 definition [6]. In addition, patients with missing data relevant to the study analysis, i.e., survival status, comorbidities, post-discharge destination, or nursing care dependency were excluded from analysis.

Following, patients were grouped using an ICD abstraction strategy to have suffered from severe infections (defined as infections without an acute organ dysfunction), sepsis (defined as infections with an acute organ dysfunction), and septic shock (defined as infections with an acute organ dysfunction and shock) compatible with the new Sepsis-3 definition [6]. ICD-10-GM codes and Operation and Procedure (OPS) codes of the German Institute for Medical Documentation that were used to identify patients with acute organ dysfunction are set forth in S2 Table. The accuracy of our ICD-abstraction strategy to comply with the Sepsis-3 definition [6], was validated by an additional manual chart review using our hospital data records (S3 Table). Hospital survivors were followed up for 5 years from the day of their qualifying hospital admission or, whatever occurred first, until June 30, 2018, disenrollment from health care insurance (e.g., change of health care insurer), or death.

### Data source

Data were obtained from the Knappschaft Krankenkasse claims database, a large German statutory health care insurer with approximately 1.8 million insurants, which means 3% of all statutory health insured Germans accordingly. All claims can be linked using unique member identifiers and arrayed in chronological order to provide a detailed longitudinal profile of all medical and pharmacy services used by each patient. The database consists of received outpatient medications, facilities, and professional medical services. The database also includes member demographics, vital status information, inpatient and outpatient diagnoses (in the format of ICD, 10^th^ revision, German modification), inpatient and outpatient procedures (in the format of ICD-10-GM and OPS codes), degree of nursing care dependency (legal definition according to the first German Pflegestärkungsgesetz of January 1, 2015), and the Case Mix Index (CMI). The CMI is a relative value assigned to a diagnosis-related group in the German health care system, based on the medical conditions and procedures (diagnosis, procedures, age), complexity (comorbidity), and other needs. Thus, the CMI is meant to indicate amount of resources required to treat a given patient. In addition, all patients were categorized according to their etiology of infection that was derived from related ICD coding of the index admission.

### Statistical analysis

Continuous variables are presented as means ± standard deviation (SD) in case of normal distribution and as median and interquartile range (25th; 75th percentile) in case of non-normally distributed variables. Categorical variables were characterized by numbers (and percentages) and compared using the Chi-square test. Continuous variables were compared by two-way analysis of variance (including Games-Howell`s post hoc test) for parametric variables, and the Kruskal-Wallis-test (followed by the post hoc Dunn test) for non-parametric variables, as appropriate. Due to the exploratory character of our study no attempt was made to adjust for multiplicity. Odds ratios were calculated by the matched Mantel Haenszel’s estimation (ratio of discordant pairs). Patients were censored as of the date of disenrollment or June 30, 2018, whichever occurred first. In this context, 26.6% (648/2440) after septic shock, 22.4% (11905/53085) after sepsis, and 25.0% (7103/28449) after severe infections were lost to the 5-year follow up.

**Fig 2:**
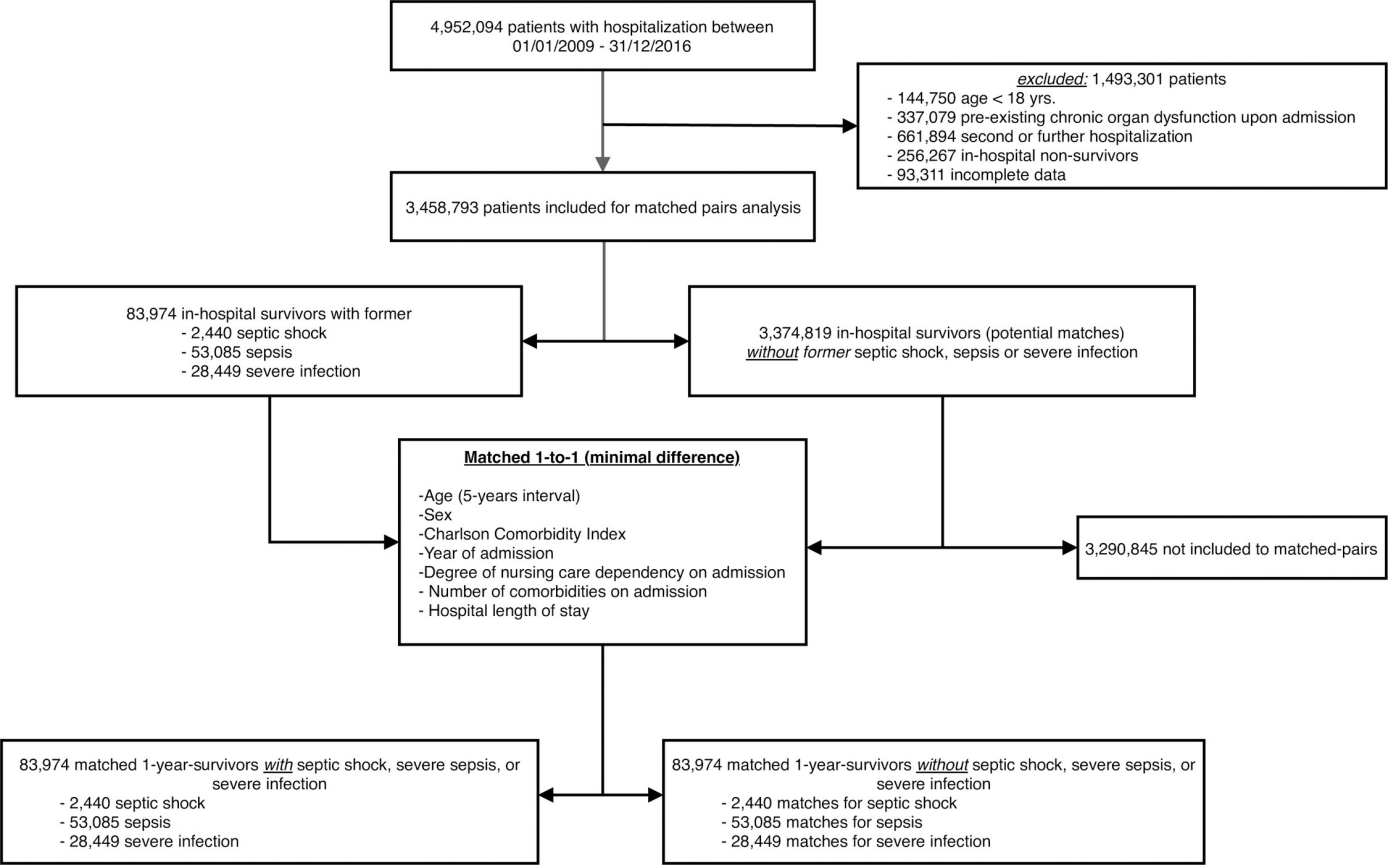
Flow chart showing selection of patients for matched analyses, shown for in-hospital survivors and non-survivors.

For matched pairs analysis we used a one-to-one (1:1) exact matching approach without replacement as described in Fig 2.

Following, to compare patients discharged to home and self-care or to rehabilitation facilities a second exact matching (1:1) procedure was performed using the same matching criteria as mentioned above.

Survival probabilities were graphically assessed by the Kaplan–Meier method and compared using a two-sided log-rank test. We used a univariate Cox regression to determine a crude hazard ratio (HR_crude_) of patients with former septic shock, sepsis, and severe infections without organ dysfunction compared to their matched pairs. Pertinent confounders were determined in accordance to our baseline covariates with a standardized difference of more than 10% indicating an insufficient covariate balance and evidence from previous studies regarding prespecified risk factors [11, 13, 15]. Thereafter, we used a multivariate Cox regression analysis to determine an adjusted hazard ratio (HR_adj_) with age, sex, Charlson Comorbidity index, degree of nursing care dependency, hospital length of stay, comorbid conditions, and Case Mix Index as covariates.

Additionally, multivariate Cox regression analysis (adjusted for age, sex, Charlson Comorbidity index prior to sepsis, comorbid conditions, degree of nursing care dependency, etiology of infection, hospital length of stay, site of organ dysfunction, and Case Mix Index as covariates) was used to compare the survival of exactly matched patients discharged to home or self-care or specialized rehabilitation facilities with former severe infections, sepsis, or septic shock.

## Results

### Patient characteristics

We identified a total of 83,974 hospital survivors with former severe infections (n = 28,449), sepsis (n = 53,085), and septic shock (n = 2,440) between January 1, 2009 and December 31, 2016 (Fig 1; Table 1).

**Table 1:** Characteristics of patients with former septic shock, sepsis or severe infections without organ dysfunction each compared to matched controls.

Variable	Septic shock (n = 2,440)	Controls (n = 2440)	P-value	Sepsis (n = 53,085)	Controls (n = 53,085)	P-value	Severe infections (n = 28,449)	Controls (n = 28,449)	P-value
Matched characteristics									
Age yrs. (±SD)	75.1 (±10.9)	75.1 (±10.9)	0.999	78.3 (±10.5)	78.2 (±10.5)	0.515	75.4 (12.6)	75.4 (±12.6)	0.875
Male sex n,(%)	1427 (58.5%)	1427 (58.5%)	1.000	27579 (52.0%)	27579 (52.0%)	1.000	14753 (51.9%)	14753 (51.9%)	1.000
CCI prior to event (±SD)	4.6 (±3.3)	4.6 (±3.3)	0.997	4.8 (±3.3)	4.8 (±3.3)	0.934	4.4 (±3.4)	4.4 (±3.4)	0.934
No of comorbidities	2.4 (± 2.3)	2.4 (±2.2)	0.765	2.6 (± 2.4)	2.6 (±2.3)	0.402	2.0 (± 2.1)	2.0 (±2.2)	0.702
Degree of nursing care dependency at admission, n (%)			0.991			0.986			
- none	1486 (60.9%)	1489 (61.0%)		27472 (51.8%)	27491 (51.8%)		16158 (56.8%)	16184 (56.9%)	
- 1	442 (18.1%)	441 (18.1%)		12117 (22.8%)	12119 (22.8%)		5115 (18.0%)	5113 (18.0%)	
- 2	400 (16.4%)	401 (16.4%)		10746 (20.2%)	7367 (20.2%)		5267 (18.5%)	5270 (18.5%)	
- 3	112 (4.6%)	109 (4.5%)		2750 (5.2%)	2728 (5.1%)		1909 (6.7%)	1882 (6.6%)	
Year of admission, n (%)			1.000			1.000			1.000
- 2009	207 (8.5%)	207 (8.5%)		5279 (9.9%)	5279 (9.9%)		3480 (12.2%)	3480 (12.2%)	
- 2010	254 (10.1%)	254 (10.1%)		5447 (10.3%)	5447 (10.3%)		3355 (11.8%)	3355 (11.8%)	
- 2011	288 (11.8%)	288 (11.8%)		5638(10.6%)	5638(10.6%)		3268(11.5%)	3268(11.5%)	
- 2012	261 (10.7%)	261 (10.7%)		6072 (11.4%)	6072 (11.4%)		3332 (11.7%)	3332 (11.7%)	
- 2013	320 (13.1%)	320 (13.1%)		6450 (12.2%)	6450 (12.2%)		3455 (12.1%)	3455 (12.1%)	
- 2014	346 (14.2%)	346 (14.2%)		7088 (13.4%)	7088 (13.4%)		3802(13.4%)	3802(13.4%)	
- 2015	378 (15.5%)	378 (15.5%)		8202 (15.4%)	8202 (15.4%)		3808 (13.4%)	3808 (13.4%)	
- 2016	392 (16.1%)	392 (16.1%)		8909 (16.8%)	8909 (16.8%)		3949(13.9%)	3949(13.9%)	
Hospital LOS, days (IQR)	15 (6;31)	15 (6;30)	0.981	15 (7;26)	15 (6;26)	0.936	11 (7;20)	11 (7;21)	0.964
**Unmatched characteristics**									
Comorbid condition, n (%)									
- COPD	660 (27.1%)	1742 (24.1%)	<0.001	14013 (26.4%)	13031 (24.5%)	<0.001	6252 (22.0%)	6427 (22.6%)	0.061
- Ischemic heart disease	1061 (43.5%)	3142 (43.5%)	0.973	25150 (47.4%)	24714 (46.6%)	0.973	11497 (40.4%)	12117 (42.6%)	<0.001
- Diabetes mellitus	1112 (45.6%)	3220 (44.5%)	0.216	26167 (49.3%)	24694 (46.5%)	0.001	12564 (44.2%)	12175 (42.8%)	<0.001
- Malignant—neoplasms	693 (28.4%)	2366 (32.7%)	<0.001	14057 (26.5%)	17643 (32.8%)	<0.001	8667 (30.5%)	8840 (31.1%)	0.094
- None of the above	449 (20.5%)	1442 (19.9%)	0.431	9739 (18.3%)	9606 (18.1%)	0.199	6283 (22.1%)	6230 (21.9%)	0.564
- Case Mix Index (±SD)	8.4 (±10.4)	1.5 (±1.7)	<0.001	4.3 (±7.1)	1.3 (±1.4)	<0.001	2.4 (±4.1)	1.3 (±1.4)	<0.001
***Time-dependent risk of 5-year mortality in matched hospital survivors***									
Crude hazard ratio (HR_crude_)	1.70 (1.57–1.84)	1.00 (reference)	<0.001	1.66 (1.63–1.69)	1.00 (reference)	<0.001	1.59 (.54–1.63)	1.00 (reference)	<0.001
Adjusted hazard ratio (HR_adj_)*	2.03 (1.87–2.19)	1.00 (reference)	<0.001	1.73 (1.71–1.76)	1.00 (reference)	<0.001	1.70 (1.65–1.74)	1.00 (reference)	<0.001

Data are presented as n (%); means (±SD); median, IQR: interquartile ranges (25th, 75th percentile), hazard ratios were provided with corresponding 95%-CI; CCI: Charlson Comorbidity Index, LOS: length of stay; COPD: Chronic obstructive pulmonary disease; degree 1 of nursing care dependency: significant; degree 2 of nursing care dependency: severe; degree 3 of nursing care dependency: heaviest.

* Covariates included in the multivariable-adjusted Cox regression model were age, sex, Charlson Comorbidity index, degree of nursing care dependency, hospital length of stay, comorbid conditions, and Case Mix Index.

Most study subjects were over 65 years old (septic shock: 84.9%, sepsis: 89.5%, severe infections: 85.0%). 52.3% (61,335/117,202) of patients were male with a statistically significant higher proportion of males in septic shock patients (58.5%, *P*<0.001).

The most common causes of infection were pneumonia (septic shock: 37.8%, sepsis: 37.2%, severe infections: 19.6%) and urinary tract infections (septic shock: 8.6%, sepsis: 29.7%, severe infections: 22.7%). Furthermore, there was a greater rate of abdominal (13.4%) and central line infections (6.2%) in septic shock patients compared to sepsis (5.3% and 3.4%, respectively) and severe infections (3.9% and 3.0%, respectively).

### Long-Term mortality

**Fig 3:**
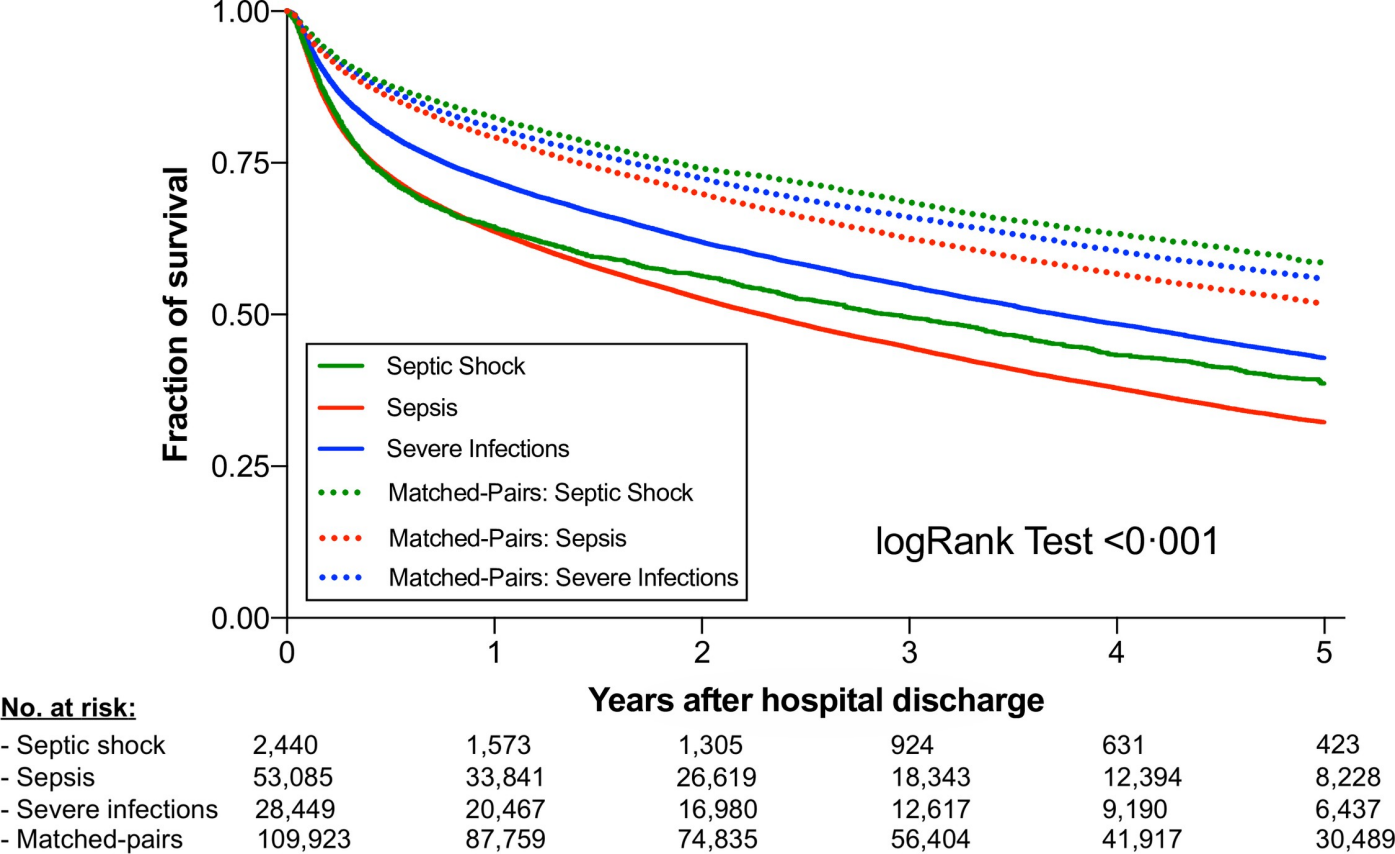
5-year survival of septic shock, sepsis and severe infections compared to matched pairs. Shown are hospital survivors after suffering from septic shock (green), sepsis (red), or severe infections without organ dysfunction (blue) compared to matched patients with hospitalizations without infection. Kaplan-Meier estimates were used to calculate probabilities of 5-year survival based on the above-mentioned classification. 5-year survival was markedly and significantly decreased in patients having suffered from septic shock, sepsis, and severe infections.

The 5-year mortality of hospital survivors were 56.1% (1369/2440) with septic shock, 62.1% (32952/53085) with sepsis, and 52.4% (14909/28449) with severe infections (Fig 3).

Time-dependent Cox regression revealed a significantly greater mortality risk within the 5-year observation period in patients having suffered from severe infections (HR_adj_ 1.70, 95%-CI 1.65 to 1.74; *P*<0.001), sepsis (HR_adj_ 1.73, 95%-CI 1.71 to 1.76; *P*<0.001), or septic shock (HR_adj_ 2.03, 95%-CI 1.87 to 2.19; *P*<0.001) compared to matched controls (Table 1).

### Medical care charges and long-term outcome

One year after the index admission, mean cumulative in-patient medical care charges of hospital survivors paid by the health care insurer averaged €10,479 (±17,190) for septic shock, €8,208 (±17,190) for sepsis, and €7,244 (±16,006) for infections cases.

On average, patients after having suffered from septic shock, sepsis, and severe infections revealed 7.4 (18,118/2,440), 7.2 (382,489/53,085), and 6.8 (190,858/28,449) new health-related diagnoses within the first year after hospital discharge, respectively. In addition, the odds ratio of being assigned to a higher level of care dependency within the first year after index hospitalization were 7.49 (95%-CI 5.97 to 9.39) in septic shock, 6.86 (95%-CI 6.53 to 7.21) in sepsis, and 6.84 (95%-CI 6.38 to 7.33) in severe infections compared to their matched controls.

### Importance of aftercare

**Fig 4:**
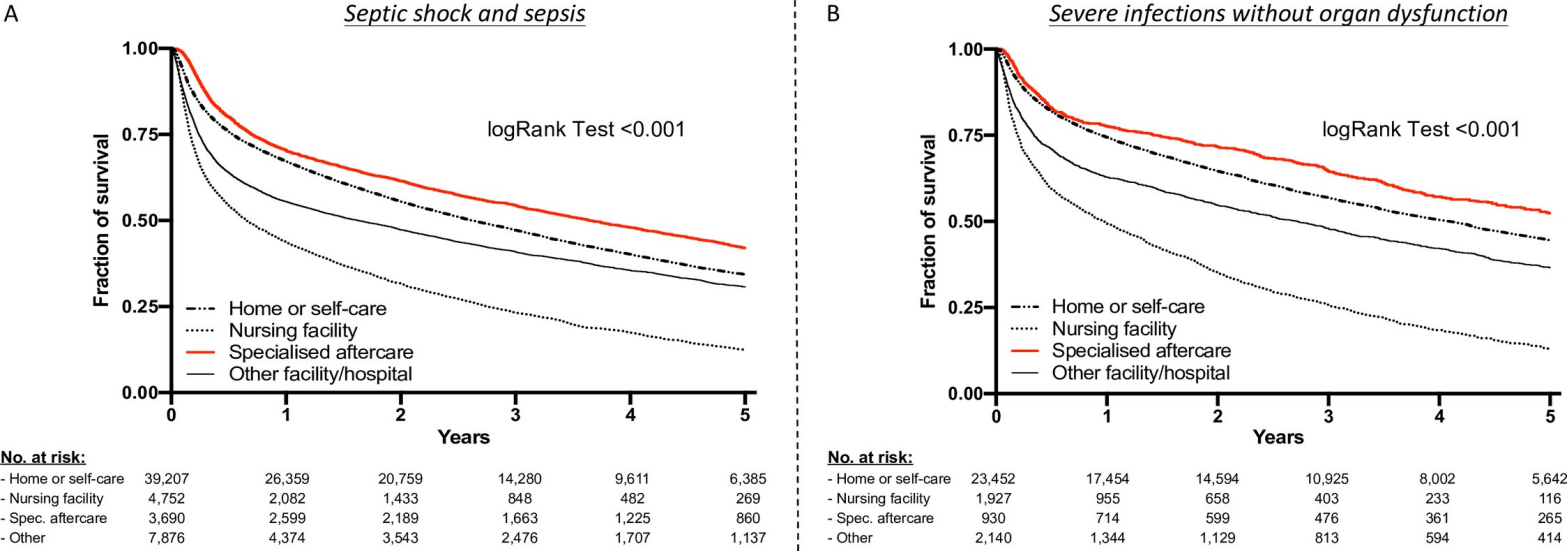
**5-year survival of A) septic shock and sepsis and B) severe infections without organ dysfunction stratified for their discharge destination.** Unmatched hospital survivors are included in analysis. Kaplan-Meier estimates were used to calculate probabilities of 5-year survival based on discharge to home or self-care, nursing care facility, rehabilitation facility, and other facilities. 5-year survival was significantly greater in patients transferred to rehabilitation facilities.

Surprisingly, most survivors were discharged home or to self-care (septic shock 53.1%, sepsis 71.1%, severe infections 82.4%), and only 14.5% of survivors of septic shock, 6.3% of those having suffered from sepsis, and 3.3% of those having suffered from severe infections were transferred to a post-discharge rehabilitation. However, the lowest 5-year mortality rates were found in patients with former sepsis or septic shock (53.4%; 1971/3960) discharged to a rehabilitation facility compared to patients discharged to home or self-care (59.6% (23381/39207). The same was true in patients with former severe infections without organ dysfunction (rehabilitation: 42.4%, 394/930 vs. home/self-care: 50.6%, 11878/23451; Fig 4).

Following, we compared exactly matched patients discharged to a rehab facility with patients discharged to home and self-care additionally (Table 2 and Table 3).

**Table 2:** Characteristics of matched patients with sepsis and septic shock stratified according discharge destination after index admission.

Variable	Home or self-care n = 3690	Rehabilitation facility n = 3690	P-value
Age *yrs*. (range/±SD)	74.6 (18-101/±10.6)	74.6 (19-100/±10.5)	0.935
Male sex (%)	2182 (59.1%)	2182 (59.1%)	1.000
Charlson Comorbidity Index prior sepsis (±SD)	3.74 (±3.01)	3.75 (±3.02)	0.975
Comorbid condition (%)			
- Chronic obstructive pulmonary disease	841 (22.8%)	832 (22.6%)	0.802
- Ischemic heart disease	1573 (42.6%)	1642 (44.5%)	0.105
- Diabetes mellitus	1630 (44.2%)	1709 (46.3%)	0.065
- Malignant neoplasm	850 (23.0%)	791 (21.4%)	0.099
- None of the above	909 (24.6%)	856 (23.2%)	0.148
Degree of nursing care dependency before admission (%)			0.992
- none	2941 (79.7%)	2940 (79.7%)	
- 1	509 (13.8%)	510 (13.8%)	
- 2	219 (5.9%)	217 (5.9%)	
- 3	21 (0.6%)	23 (0.6%)	
Etiology of infection (%)			<0.001
- Pneumonia	1178 (31.9%)	1589 (43.1%)	
- Urinary tract infection	1104 (29.9%)	880 (23.9%)	
- Abdominal infection	259 (7.0%)	182 (4.9%)	
- Skin or muscle infection	100 (2.7%)	87 (2.4%)	
- Line infection	136 (3.7%)	326 (8.8%)	
- Other / Unknown origin	913 (24.7%)	626 (17.0%)	
Hospital length of stay			<0.001
- median [days] (IQR)	17 (10;29)	33 (21;49)	
- <1 week	314 (8.5%)	47 (1.3%)	
- 1–4 weeks	2425 (65.7%)	1466 (39.7%)	
- 5–10 weeks	829 (22.5%)	1820 (49.3%)	
- >10 weeks	122 (3.3%)	357 (9.7%)	
Site of organ system with dysfunction			
- Respiratory	1182 (32.0%)	1935 (52.4%)	<0.001
- Central nervous system	807 (21.9%)	1432 (38.8%)	<0.001
- Cardiovascular	1566 (42.4%)	1858 (50.4%)	<0.001
- Hepatic	49 (1.3%)	72 (2.0%)	0.035
- Coagulation	34 (0.9%)	69 (1.9%)	<0.001
- Renal	2383 (64.6%)	1984 (53.8%)	<0.001
Case Mix Index (±SD)	4.09 (±6.87)	11.19 (±11.85)	<0.001
***Time-dependent risk of 5-year mortality in matched hospital survivors with former sepsis and septic shock***			
Crude hazard ratio (HR_crude_)	1.00 (reference)	0.85 (95%-CI:0.80–0.91)	<0.001
Adjusted hazard ratio (HR_adj_)*	1.00 (reference)	0.81 (95%-CI:0.77–0.85)	<0.001

Data are presented as n (%). mean (±SD). median. IQR: interquartile ranges (25th. 75th percentile); degree 1 of nursing care dependency: significant; degree 2 of nursing care dependency: severe; degree 3 of nursing care dependency: heaviest. Only exactly (1:1) matched hospital survivors are included in calculation.

* Covariates included in the multivariable-adjusted Cox regression model were age, sex, Charlson Comorbidity index prior to sepsis, comorbid conditions, degree of nursing care dependency, etiology of infection, hospital length of stay, site of organ dysfunction and Case Mix Index.

**Table 3:** Characteristics of matched patients with severe infections without organ dysfunction stratified according discharge destination after index admission.

Variable	Home or self-care n = 930	Rehabilitation facility n = 930	P-value
Age *yrs*. (range/±SD)	73.8 (21-98/±11.2)	73.8 (20-101/±11.2)	0.955
Male sex (%)	521 (56.0%)	521 (56.0%)	1.000
Charlson Comorbidity Index prior sepsis (±SD)	3.57 (±2.87)	3.59 (±2.88)	0.930
Comorbid condition (%)			
- Chronic obstructive pulmonary disease	202 (21.7%)	233 (25.1%)	0.090
- Ischemic heart disease	348 (37.4%)	393 (42.3%)	0.033
- Diabetes mellitus	395 (42.5%)	392 (42.2%)	0.888
- Malignant neoplasm	254 (27.3%)	212 (22.8%)	0.025
- None of the above	226 (24.3%)	234 (25.2%)	0.667
Degree of nursing care dependency before admission (%)			1.000
- none	723 (77.7%)	723 (77.7%)	
- 1	130 (14.0%)	130 (14.0%)	
- 2	65 (7.0%)	65 (7.0%)	
- 3	12 (1.3%)	12 (1.3%)	
Etiology of infection (%)			<0.001
- Pneumonia	177 (19.0%)	246 (26.5%)	
- Urinary tract infection	216 (23.2%)	165 (17.7%)	
- Abdominal infection	46 (5.0%)	35 (3.8%)	
- Skin or muscle infection	15 (1.6%)	27 (2.9%)	
- Line infection	8 (0.9%)	63 (6.8%)	
- Other / Unknown origin	468 (50.3%)	394 (42.4%)	
Hospital length of stay			<0.001
- median [days] (IQR)	12 (8;19)	26 (16;42)	
- <1 week	146 (15.7%)	38 (4.1%)	
- 1–4 weeks	673 (72.4%)	471 (50.7%)	
- 5–10 weeks	102 (11.0%)	346 (37.2%)	
- >10 weeks	9 (1.0%)	75 (8.1%)	
Case Mix Index (±SD)	2.04 (±2.76)	7.25 (±9.42)	<0.001
***Time-dependent risk of 5-year mortality in matched hospital survivors with former severe infection without organ dysfunction***			
Crude hazard ratio (HR_crude_)	1.00 (reference)	0.76 (95%-CI:0.68–0.88)	<0.001
Adjusted hazard ratio (HR_adj_)*	1.00 (reference)	0.81 (95%-CI:0.73–0.90)	<0.001

Data are presented as n (%). mean (±SD). median. IQR: interquartile ranges (25th. 75th percentile); degree 1 of nursing care dependency: significant; degree 2 of nursing care dependency: severe; degree 3 of nursing care dependency: heaviest. Only exactly (1:1) matched hospital survivors are included in calculation

* Covariates included in the multivariable-adjusted Cox regression model were for age, sex, Charlson Comorbidity index prior to sepsis, comorbid conditions, degree of nursing care dependency, etiology of infection, hospital length of stay, site of organ dysfunction and Case Mix Index.

Strikingly, also these patients treated in rehabilitation facilities showed a dramatically lower 5-year mortality-risk compared to patients discharged to home or self-care after suffering from sepsis or septic shock (HR_adj_ 0.81, 95%-CI 0.77 to 0.85; *P*<0.001) as well as severe infections without organ dysfunction (HR_adj_ 0.81, 95%-CI 0.73 to 0.90; *P*<0.001).

## Discussion

The main findings of this study are: 1) Long-term mortality and morbidity were markedly increased for (at least) 5 years in hospital survivors of septic shock, sepsis and severe infections without organ dysfunction; 2) highest survival rates were observed in patients transferred to rehabilitation facilities.

Our study in a large retrospective cohort sheds light on long-term mortality, outcome, and importance of aftercare in hospital survivors with former severe infections without organ dysfunction, sepsis, and septic shock closely matched to the Sepsis-3 definition [6]. Therefore, our findings provide hitherto missing results and complement prior studies regarding long-term outcome [12, 19–22].

Our data are in line with smaller studies suggesting that sepsis also impacts long-term mortality [21–23]. We could confirm that sepsis and septic shock are worse regarding impact on the patients’ long-term outcome compared to non-infectious illnesses. These findings are in line with the population-based study of Henriksen and colleagues demonstrating an almost 2-fold greater mortality compared with sepsis-free individuals [22]. Strikingly, we elucidated that survivors of severe infections that may not be covered by Sepsis-3 definition were also affected by higher mortality risk with an estimated HR of 1.70 for the 5-year observation period. In this regard, our results corroborate the findings of Honselmann and colleagues [12] reporting a comparable 1-year mortality in pneumonia and septic patients as well as the findings of Henriksen and colleagues who found that sepsis severity (comparing sepsis, severe sepsis, and septic shock as defined by Sepsis-1 criteria) had no additional impact on 5-year survival [22].

The same was true, but even to a larger extent with odds ratios of approximately seven for the degree of nursing care dependency and new health related diagnoses within the first year after discharge from index-hospitalization in all three entities. Taking these aspects into account, acute organ dysfunction may not to be the only decisive predictor regarding long-term outcome in severe infections [24].

The poor prognosis as described in our study for all three groups raises the question whether a specific aftercare after discharge from hospital can improve these patients’ outcome. Most studies of critical ill patients have examined the outcomes of acute rehabilitation on ICUs [25, 26] or immediately post-ICU hospital-based rehabilitation [27] that were not associated with a convincing benefit. However, there is paucity of clinical trial evidence for post-discharge rehabilitation treatment, among other aspects, because these studies have not stringently examined hard clinical endpoints such as mortality [28, 29]. Despite this insufficient evidence, experts recommend post-discharge referral to physical therapy to improve exercise capacity, strength, and independent completion of activities of daily life [30]. This recommendation is supported by one nationwide, population-based cohort study from Taiwan that showed a risk reduction of 10-year mortality by referral to rehabilitation, but not stratifying infections according organ dysfunctions [19]. In our cohort, we also found a higher 5-year survival of sepsis patients when transferred to rehabilitation facilities were greater compared to those discharged to other destinations. The same was true for patients suffering from severe infections without organ dysfunctions that may not be covered by Sepsis-3 definition. Hence, our results can provide a rationale for a rigorous approach with more attention to post-acute care in patients having suffered from both sepsis and severe infections.

Currently, only a small percentage of surviving patients were transferred to a specialized rehabilitation facility, consistent with other studies reporting that sepsis survivors are less frequently transferred to rehabilitation facilities than patients with other diagnoses [19, 31]. Possibly, the burden carried by formerly septic patients is less appreciated outside the intensive care community and / or the logistics of transfer from an ICU to rehabilitation care does not work properly [30, 32]. Accordingly, current standard practices may be suboptimal and our data suggest that in-hospital survivors from sepsis and septic shock may benefit from a more intense post-discharge care management including a “sepsis-specific” aftercare, as suggested and investigated by the Smooth Study Group [33]. However, this hypothesis must be investigated in an independent prospective study to get a clear and unequivocal rationale for specialized aftercare and overcome the major limitations of this retrospective study.

### Limitations

In addition to the retrospective character, our study has some further limitations that should be noted here. First, we used an ICD abstraction strategy based on administrative data to identify patients with septic shock, sepsis, or severe infections without organ dysfunction. Since the Sepsis-3 definition is based on clinical criteria condensed into the SOFA score a potential bias and confounding due to using claim data may occur. Unfortunately, potentially important clinical variables (e.g., drugs or the SOFA score) during hospitalization are not part of the claim data base. Thus, residual confounding could still exist despite utmost efforts to avoid relevant bias, which could limit data reproducibility. Accordingly, we cannot entirely clarify to what extent these variables influenced outcome. However, a subsequent independent analysis revealed an accuracy of more than 90% suggesting a sufficient classification by our abstraction strategy. Importantly, this does not absolve from the obligation to investigate this association in prospective randomized controlled studies for plausibility as our results can only provide a rationale for such a study. Second, our results may be affected by changing patterns of physician’s diagnosis and coding practice over time which could impact on the results of our ICD abstraction strategy. However, since the ICD-10-GM classification model has been in place since 2009 we can assume that no major changes in the coding practices occurred during the time of this study. Third, only adults in German hospitals were included and data are thus not applicable to children or to other countries, especially low-income countries. Fourth, the medical claim database does not contain information about several important clinical factors such as body mass index, more detailed severity of illness (e.g., SOFA score), and laboratory findings, and thus a priori susceptible to a biased patient selection and restricted patient stratification.

### Conclusions

Long-term mortality and morbidity of hospital survivors are markedly increased in patients following severe infections and sepsis. Furthermore, the greatest survival rates were recorded in patients discharged to rehabilitation facilities. Hence, our results emphasize that sepsis-related aftercare programs may relevantly improve prognosis of septic patients. In addition patients with severe infection, which potentially do not meet Sepsis-3 criteria, could also benefit from structured aftercare programs. In conclusion, the results of our studies warrant further investigation regarding causal relations in prospective randomized controlled trials.

## Supporting information

S1 Table ICD-10-GM codes related to an infection for case selection. (PDF)

S2 Table ICD-10-GM and OPS codes related to acute organ dysfunction and shock. (PDF)

S3 Table Validation of ICD-/OPS-code abstraction procedure (implicit sepsis diagnosis) using medical record review for case stratification according Sepsis-3 definition. (PDF)

S1 File Source data file. (XLSX)

S2 File STROBE checklist. (PDF)
