# Supplementary material for: Long-term mortality and outcome in hospital survivors of septic shock, sepsis, and severe infections: The importance of aftercare
Source: PLoS One. 2020 Feb 12;15(2):e0228952. doi: 10.1371/journal.pone.0228952 (PMC7015408; doi:10.1371/journal.pone.0228952)
Supplement: S1 Table — (PDF) [file pone.0228952.s001.pdf]

S1 Table: ICD-10-GM codes related to an infection for case selection

ICD-10-GM code	ICD-10-GM code description
**I - Certain infectious and parasitic diseases**	
A00-A09	Intestinal infectious diseases
A15-A19	Tuberculosis
A20-A28	Certain zoonotic bacterial diseases
A30-A49	Other bacterial diseases
A50-A64	Infections with a predominantly sexual mode of transmission
A70-A74	Other diseases caused by chlamydia
A75-A79	Rickettsioses
A92-A99	Arthropod-borne viral fevers and viral hemorrhagic fevers
B00	Herpes viral [herpes simplex] infections
B01	Varicella [chickenpox]
B02	Zoster [herpes zoster]
B05	Measles
B06	Rubella [German measles]
B15-B19	Viral hepatitis
B25-B34	Other viral diseases
B37	Candidiasis
B38	Coccidioidomycosis
B39	Histoplasmosis
B40	Blastomycosis
B44	Aspergillosis
B49	Unspecified mycosis
B50-B64	Protozoal diseases
B99	Other infectious diseases
**VI - Diseases of the nervous system**	
G00	Bacterial meningitis, not elsewhere classified
G01	Meningitis in bacterial diseases classified elsewhere
G02	Meningitis in other infectious and parasitic diseases classified elsewhere
G04	Encephalitis, myelitis and encephalomyelitis
G05	Encephalitis, myelitis and encephalomyelitis in diseases classified elsewhere
G06	Intracranial and intraspinal abscess and granuloma
G07	Intracranial and intraspinal abscess and granuloma in diseases classified elsewhere
G08	Intracranial and intraspinal phlebitis and thrombophlebitis
**VII - Diseases of the eye and adnexa**	
H05.0	Acute inflammation of orbit
H44.0	Purulent endophthalmitis
**VIII - Diseases of the ear and mastoid process**	
H60.2	Malignant otitis externa
H70.0	Acute mastoiditis
**IX - Diseases of the circulatory system**	
I30.1	Infective pericarditis
I33.0	Acute and subacute infective endocarditis
I40.0	Infective myocarditis
**X - Diseases of the respiratory system**	
J01	Acute sinusitis
J02	Acute pharyngitis
J03	Acute tonsillitis
J04	Acute laryngitis and tracheitis
J06	Acute upper respiratory infections of multiple and unspecified sites
J09-J18	Influenza and pneumonia
J20-22	Other acute lower respiratory infections
J36	Peritonsillar abscess

ICD-10-GM code	ICD-10-GM code description
J39.0	Retropharyngeal and parapharyngeal abscess
J39.1	Other abscess of pharynx
J44.0	Chronic obstructive pulmonary disease with acute lower respiratory infection
J47.0	Bronchiectasis
J85-J86	Suppurative and necrotic conditions of lower respiratory tract
J98.50	Mediastinitis
**XI - Diseases of the digestive system**	
K10.2	Inflammatory conditions of jaws
K11.3	Abscess of salivary gland
K12.2	Cellulitis and abscess of mouth
K35	Acute appendicitis
K57.0	Diverticular disease of small intestine with perforation and abscess
K57.2	Diverticular disease of large intestine with perforation and abscess
K57.4	Diverticular disease of both small and large intestine with perforation and abscess
K57.8	Diverticular disease of intestine, part unspecified, with perforation and abscess
K61	Abscess of anal and rectal regions
K63.0	Abscess of intestine
K63.1	Perforation of intestine (nontraumatic)
K65	Peritonitis
K75.0	Abscess of liver
K75.1	Phlebitis of portal vein
K81.0	Acute cholecystitis
K83.0	Cholangitis
**XII - Diseases of the skin and subcutaneous tissue**	
L00	Staphylococcal scalded skin syndrome
L01	Impetigo
L02	Cutaneous abscess, furuncle and carbuncle
L03	Cellulitis
L04	Acute lymphadenitis
L05	Pilonidal cyst
L08	Other local infections of skin and subcutaneous tissue
L88	Pyoderma gangrenosum
**XIII - Diseases of the musculoskeletal system and connective tissue**	
M00	Pyogenic arthritis
M01	Direct infections of joint in infectious and parasitic diseases classified elsewhere
M46.2	Osteomyelitis of vertebra
M46.3	Infection of intervertebral disc (pyogenic)
M46.5	Other infective spondylopathies
M60.0	Infective myositis
M72.6	Necrotizing fasciitis
M86	Osteomyelitis
**XIV - Diseases of the genitourinary system**	
N10	Acute tubulo-interstitial nephritis
N30.0	Acute cystitis
N30.8	Other cystitis
N30.9	Cystitis, unspecified
N39.0	Urinary tract infection, site not specified
N41.0	Acute prostatitis
N41.2	Abscess of prostate
N41.3	Prostatocystitis
N41.8	Other inflammatory diseases of prostate
N45	Orchitis and epididymitis
N70-77	Inflammatory diseases of female pelvic organs

ICD-10-GM code	ICD-10-GM code description
**XV - Pregnancy, childbirth and the puerperium**	
O03.0	Spontaneous abortion : incomplete, complicated by genital tract and pelvic infection
O03.5	Spontaneous abortion : complete or unspecified, complicated by genital tract and pelvic infection
O04.0	Medical abortion : incomplete, complicated by genital tract and pelvic infection
O05.0	Other abortion : incomplete, complicated by genital tract and pelvic infection
O06.0	Unspecified abortion : incomplete, complicated by genital tract and pelvic infection
O07.0	Failed medical abortion, complicated by genital tract and pelvic infection
O08.0	Genital tract and pelvic infection following abortion and ectopic and molar pregnancy
O23	Infections of genitourinary tract in pregnancy
O41.1	Infection of amniotic sac and membranes
O75.3	Other infection during labour
O85	Puerperal sepsis
O86	Other puerperal infections
**XVIII - Symptoms, signs and abnormal clinical and laboratory findings, not elsewhere classified**	
R57.2	Septic shock
R65.0	Systemic Inflammatory Response Syndrome of infectious origin without organ failure
R65.1	Systemic Inflammatory Response Syndrome of infectious origin with organ failure
**XIX - Injury, poisoning and certain other consequences of external causes**	
T79.3	Post-traumatic wound infection, not elsewhere classified
T82.x - T85.x	Infection and inflammatory reaction due to other cardiac and vascular devices, implants and grafts

International Classification of Diseases, 10th revision, German modification (ICD-10-GM) codes with diagnosis related to an infection used for appropriate case selection in context with the ICD abstraction strategy.
