# Supplementary material for: Long-term mortality and outcome in hospital survivors of septic shock, sepsis, and severe infections: The importance of aftercare
Source: PLoS One. 2020 Feb 12;15(2):e0228952. doi: 10.1371/journal.pone.0228952 (PMC7015408; doi:10.1371/journal.pone.0228952)
Supplement: S2 Table — (PDF) [file pone.0228952.s002.pdf]

S2 Table: ICD-10-GM and OPS codes related to acute organ dysfunction and shock

Organ system	ICD-10-GM or OPS code	ICD-10-GM or OPS code description
Cardiovascular	ICD - I50	Heart failure
	ICD - I95.8	Other hypotension
	ICD - I95.9	Hypotension, unspecified
	ICD - I98.1	Cardiovascular disorders in other infectious and parasitic diseases classified elsewhere
	ICD - I98.8	Other specified disorders of circulatory
	ICD - I99	Other and unspecified disorders of circulatory system
Hematologic	ICD - D65	Disseminated intravascular coagulation (defibrination syndrome)
	ICD - D69.5	Secondary thrombocytopenia
	ICD - D69.6	Thrombocytopenia, unspecified
Hepatic	ICD - K72.0	Acute and subacute hepatic failure
	ICD - K72.9	Hepatic failure, unspecified
	ICD - K76.8	Other specified diseases of liver
	OPS - 8-858	Extracorporeal liver replacement therapy (liver dialysis)
Neurologic	ICD - F05.0	Delirium not superimposed on dementia, so described
	ICD - F05.8	Other delirium
	ICD - F05.9	Delirium, unspecified
	ICD - R40.0	Somnolence
	ICD - R40.1	Stupor
	ICD - R40.2	Coma, unspecified
Renal	ICD - N17	Acute renal failure
	OPS - 8-853	Hemofiltration
	OPS - 8-853	Hemodialysis
	OPS - 8-854	Hemodiafiltration
Respiratory	ICD - J80	Adult respiratory distress syndrome
	ICD - J96.0	Acute respiratory failure
	ICD - U04.9	Severe acute respiratory syndrome (SARS)
	OPS - 8-852	Extracorporeal membrane oxygenation (ECMO) therapy
	OPS - 8-713	Mechanical Ventilation in adults
	OPS - 8-714	Specialized mechanical ventilation in severe respiratory failure
Other	ICD - R65.1	Severe Sepsis
Shock	ICD - R57.2	Septic shock
	ICD - R57.8	Other shock
	ICD - R57.9	Shock, unspecified

International Classification of Diseases, 10th revision, German modification (ICD-10-GM) and Operation and Procedure (OPS) codes for appropriate group allocation in septic shock, sepsis and severe infections in context with the ICD abstraction strategy.
