# Supplementary material for: Long-term mortality and outcome in hospital survivors of septic shock, sepsis, and severe infections: The importance of aftercare
Source: PLoS One. 2020 Feb 12;15(2):e0228952. doi: 10.1371/journal.pone.0228952 (PMC7015408; doi:10.1371/journal.pone.0228952)
Supplement: S3 Table — (PDF) [file pone.0228952.s003.pdf]

S3 Table: Validation of ICD-/OPS-code abstraction procedure (implicit sepsis diagnosis) using medical record review for case stratification according Sepsis-3 definition.

200 selected patients were analyzed via manual chart review using hospital data records. Patients were considered eligible and randomly selected if they were treated for sepsis or severe infections on our ICU (University Hospital Knappschaftskrankenhaus Bochum) between January 2018 and April 2019 to validate the accuracy of our ICD-abstraction strategy. For subsequent analysis 100 patients with sepsis (increase in SOFA score ≥ 2 points) and 100 patients with severe infection without sepsis (increase in SOFA score < 2points) as per Sepsis-3 definition [6] were selected and also stratified regarding our ICD abstraction classification (Table A). In addition, all septic patients (n=100) were subclassified regarding septic shock vs. sepsis in accordance to Sepsis-3 definition and our ICD abstraction classification (Table B). Results of patients' classification are set forth in Table A and Table B.

Table A:

	**Sepsis** (n) according to Sepsis-3 definition	**Severe Infections** (n) **- non septic -** according to Sepsis-3 definition	**Total** (n)
**Sepsis** (n) according to our ICD abstraction strategy	100	19	119
**Severe infection** (n) **- without organ dysfunction -** according to our ICD abstraction strategy	0	81	81
**Total** (n)	100	100	

Sensitivity: 84.03% (95%-CI: 76.19% to 90.10%)
Specificity: 100.00% (95%-CI: 95.55% to 100.00%)
PPV: 100.00% (95%-CI: n/a)
NPV: 81.00% (95%-CI: 85.56% to 94.18%)
Accuracy: 90.50% (95%-CI: 82.67% to 92.16%)

Table B:

	**Sepsis** (n) according to Sepsis-3 definition	**Septic Shock** (n) according to Sepsis-3 definition	**Total** (n)
**Sepsis** (n) according to our ICD abstraction strategy	83	1	84
**Septic shock**(n) according to our ICD abstraction strategy	4	12	16
Total (n)	87	13	

Sensitivity: 92.31% (95%-CI: 63.97% to 99.81%)
Specificity: 95.40% (95%-CI: 88.64% to 98.73%)
PPV: 75.00% (95%-CI: 53.21% to 88.78%)
NPV: 98.81% (95%-CI: 92.66% to 99.82%)
Accuracy: 95.00% (95%-CI: 88.72% to 98.36%)
